# An *ABCB11* variant registry and novel knockin mouse model of PFIC2 based on the clinically relevant *ABCB11 E297G* variant

**DOI:** 10.1016/j.jlr.2025.100840

**Published:** 2025-06-11

**Authors:** Eric L. Bell, Jennifer K. Truong, Youhwa Jo, Adrianne Kolpak, Lauren Chunn, Natalie Syverud, Melida Mahinic, Jessica R. Durrant, Eitan Hoch, Bharat Reddy, Patrick Stoiber, John P. Miller, Yong Ren, Jonathan Moore, Robert O. Hughes, Alastair S. Garfield

**Affiliations:** 1 Rectify Pharmaceuticals, Cambridge, MA, USA; 2 Genomenon, Ann Arbor, MI, USA; 3 DTR Labs, Farmers Branch, TX, USA

**Keywords:** PFIC2, Abcb11, BSEP, cholestasis, bile acid, missense

## Abstract

Progressive familial intrahepatic cholestasis type 2 (PFIC2) is a rare pediatric cholestatic liver disease caused by genetic deficiency in the bile salt export pump (BSEP, *ABCB11*). BSEP is an ATP-binding cassette transporter and the primary regulator of hepatic bile acid efflux. Loss of BSEP function in PFIC2 leads to cholestasis and intrahepatic accumulation of bile acids, the native toxicity of which drives progressive liver injury, in a manner that correlates with *ABCB11* genotype. Here, to support ongoing PFIC2 research, we present two novel translational tools, 1) a codified evidence-based catalog of published disease relevant *ABCB11* mutations and 2) a knockin mouse model of the PFIC2-associated missense variant E297G. Using a combination of AI-based indexing of the literature and manual review, we identified 476 nonbenign *ABCB11* variants in published patients with cholestatic disease, of which 240 were associated with PFIC2. Additionally, we present phenotypic validation of a novel knockin mouse model of the cholestasis-associated *ABCB11* E297G variant. Bsep^E297G^ homozygous mice recapitulate the core molecular and pathophysiological aspects of PFIC2, including perturbed Bsep processing and membrane trafficking, cholestasis, and hepatotoxicity. Moreover, and consistent with clinical data, pharmacological ileal bile acid transporter inhibition improved the cholestatic phenotype of Bsep^E297G^ mice through increased fecal bile acid excretion. Together, these tools can support clinical and translational efforts to advance understanding and treatment of PFIC2.

Bile acids (BAs) play a critical role in metabolic homeostasis by acting as detergents, signaling molecules, and providing a mechanism for cholesterol elimination. Synthesized from cholesterol in hepatocytes, and conjugated with taurine or glycine, BAs are secreted into biliary canaliculi, along with phospholipid and cholesterol, as the constitutive elements of bile (1). Bile is concentrated and stored in the gallbladder, before being released into the duodenum to facilitate the solubilization and absorption of fats and dietary vitamins. Ninety-five percent of biliary BA are reabsorbed and recycled back to hepatocytes via enterohepatic circulation. Thus, BA homeostasis is contingent upon maintaining the appropriate balance between BA synthesis, transport, and disposal and is tightly regulated by a transcriptional network of metabolic and transporter genes. Given their detergent properties, BA are inherently cytotoxic and require appropriate buffering and compartmentalization as they traverse the hepatobiliary system to the duodenum (1). ABC transporters play a critical role in maintaining bile homeostasis and management of BA toxicity. Within the canaliculi, the formation of mixed micelles from phospholipid (exported by ABCB4) and cholesterol (exported by ABCG5/8) is critical for sequestering ionizing BA monomers and protecting the biliary epithelium (1). Within hepatocytes, BA monomers must be actively transported into canaliculi to prevent accumulation and hepatoxicity. This critical hepato-protective function is fulfilled by the bile salt export pump (BSEP) encoded by the *ABCB11* gene. Indeed, disruption of BSEP expression, localization, or function will decrease BA efflux and bile flow, and promotes primary cholestatic disorders such as progressive familial intrahepatic cholestasis type 2 (PFIC2), intrahepatic cholestasis of pregnancy (ICP), and drug-induced cholestasis (2).

PFIC2 is a rare monogenic form of severe pediatric cholestasis that arises due to congenital BSEP deficiency (3). Patients with PFIC2 present with pruritis, jaundice, elevated liver functional tests and will progress to liver failure at a rate that is dependent on genotype and level of residual BSEP function (4). Complete loss of function arising from truncating *ABCB11* variants results in the most severe and rapid progressing disease, while missense variants that retain a degree of efflux capacity are associated with slower disease progression, due to the preservation of some level of enterohepatic circulation (5). However, inheritance of these missense variants in *trans* with a truncating variant reduces net BSEP capacity and engenders a more severe clinical presentation (5). To date, approximately 200 variants have been associated with PFIC2 (6) yet the majority of variants lack full characterization, limiting the interpretation of genetic tests designed to inform diagnosis and treatment. E297G and D482G are the most prevalent PFIC2-associated *ABCB11* variants in the European ancestry and are well characterized as pathogenic within the context of PFIC2 clinical presentation (7). Patients with an E297G genotype generally demonstrate good responses to current standard of care and in vitro data indicate that both variants have normal BA transport function but are incompletely glycosylated, exhibit decreased protein stability, and have reduced trafficking to the canalicular membrane (8). It is presumed that this translates into substantially decreased, but not absent, BA efflux capacity leading to cholestasis, however this has not been formally tested within a disease relevant system.

Without therapeutic intervention the majority of PFIC2 patients will progress to end-stage liver disease due to accumulation of BA and hepatoxicity, necessitating liver transplantation. Current pretransplantation therapies focus on altering the toxicity profile of liver accumulating BA (i.e., ursodeoxycholic acid) and/or interrupting enterohepatic circulation through surgical (biliary diversion) or pharmacological [ileal bile acid transporter (IBAT) inhibition] disposal of BA, to reduce overall hepatic BA load (9). However, not all patients respond adequately to these interventions and significant unmet need remains among the PFIC2 population, especially for those individuals with marginal to no residual BSEP function (4). Given the emerging association between patient genotype, phenotype, and therapeutic response (5), further translational and drug discovery initiatives will be significantly enabled by research tools that facilitate deeper understanding of the PFIC2 genetic landscape and testing of biological and pharmacological hypotheses. To this end, we present a comprehensive literature-derived catalog of clinically relevant *ABCB11* variants and supportive evidence of their pathogenicity in PFIC2, and other cholestatic disease states, to facilitate both disease diagnosis and translational research. Furthermore, our phenotypically validated Bsep^E297G^ knockin mouse line represents a novel genotype-relevant translational model of PFIC2 and the first investigation of a disease-associated variant within a translational and pathophysiological context.

## Materials and methods

### *ABCB11* variant landscape

Published variants in *ABCB11*, including single-nucleotide variants and indels, along with their corresponding references, were automatically identified and extracted from the medical and scientific literature using Mastermind, a database assembled by Genomenon, Inc. (10). This analysis included systematic review of 717 articles describing variants in *ABCB11* published on or before May 1, 2024. Each variant was standardized using the GRCh37/hg19 genome build and the canonical transcript, NM_003742.4, as well as the nomenclature guidelines set by the Human Genome Variation Society (11). These variants were then manually interpreted according to the standards set by the American College of Medical Genetics (ACMG) and Association of Molecular Pathologists (12). This interpretation process considered clinical and functional studies from the literature, population frequencies derived from gnomAD v2.1.1, computational predictions of the effect of missense variants derived from PolyPhen-2, MutationTaster2, and SIFT, and computational predictions of splicing defects for single nucleotide variants derived from dbscSNV (13).

### Mouse model generation

CRISPR/Cas9-mediated gene editing was used to generate a constitutive knockin of the E297G variant in exon 9 of the *Abcb11* locus (NCBI transcript NM_021022.3). Additionally, a silent BmsI restriction site was inserted for the purpose of genotyping. The Cas9 protein with 5′-TGCTTTTGGTGGTGAGAATAAGG-3′ guide RNA was injected into C57BL/6NTac zygotes to generate F0 founders. Founders were screened for on-target edits by PCR (forward 5′- CACACCACCTGTTCATTTTGTTAG-3′ and reverse 5′- GGGAGCTCATACCTTTCAGTCTAG-3′) BsmI digest. Genotyping results were confirmed by sequencing clones from PCR products. Selected F0 male founder was used for in vitro fertilization to generate F1 animals that were screened for the presence of on-target edits and predicted off-target edits. All model development was contracted to Taconic Biosciences.

### Study cohorts

All mouse experiments were performed at Melior Discovery. Animals were fasted 4 h prior to biweekly tail sampling and overnight prior to termination. The deeper characterization of BA composition and the pharmacology study used female mice because they had a more severe phenotype as measured by serum total BA. The animals used in the pharmacology study were fed chow with or without 0.01% A4250 (Pharmaron) for 14 days. All experiments were conducted in accordance with the National Institutes of Health regulations of animal care covered in the Principles of Laboratory Animal Care (National Institutes of Health, 2011) and were approved by the Institutional Animal Care and Use Committee.

### Serum and biliary chemistry

Serum alanine aminotransferase (ALT) activity levels were measured using the Alanine Aminotransferase Activity Assay Kit (Sigma, Catalog No. MAK052) according to manufacturer’s instruction after diluting 20 μl of serum 1:10. Serum alkaline phosphatase (ALP) activity levels were measured using the Alkaline Phosphatase Assay Kit (Sigma, Catalog No. MAK447) according to manufacturer’s instructions by diluting 50 μl of serum 1:40. Biliary total BAs were measured using The Bile Acids Test Kit (Diazyme, Catalog No. DZ042A) and the Total Bile Acids Calibrator Set (Diazyme, Catalog No. DZ042A-CAL) according to manufacturer’s instructions with 4 μl of a final 1:1000 dilution of gallbladder bile in the assay. Biliary phospholipids were measured using the Phospholipid Assay Kit (Sigma, Catalog No. MAK122) according to manufacturer’s instructions with 20 μl of a final 1:500 dilution of gallbladder bile. Bile cholesterol levels were measured using the FUJIFILM Medical Systems USA Cholesterol E kit (Thermo Fisher Scientific, Catalog No. NC9138103) with 3 μl of gallbladder bile diluted 1:20 in 300 μl of reaction buffer.

### Serum and liver BA

Longitudinal TBA levels were measured using the Total Bile Acids Assay Kit (Diazyme, Catalog No. DZ042A) according to manufacturer’s instructions. Four microliters of undiluted serum samples were used for the analysis, and the standard curve was generated using the Total Bile Acids Calibrator Set (Diazyme, Catalog No. DZ042ACAL). BA concentrations from all liver samples were determined using AbsolutelDQ® Bile acids kit, a highly selective reversed phase and LC-MS/MS analysis at Biocrates (Innsbruck, Austria). Samples were extracted by dried filter spot technique in 96-well plate format and measured by LC-MS/MS using a 4000 QTRAP® instrument (AB Sciex, Darmstadt, Germany) with an electrospray ionization (ESI) source in negative ion multiple reaction monitoring. For highly accurate quantification, 7-point external calibration curves and ten stable isotope-labeled internal standards were used. Data were quantified using appropriate mass spectrometry software (Sciex Analyst®) and imported into Biocrates WeblDQ software for further analysis.

### RNA-seq

RNA-sequencing (RNA-seq) libraries were prepared by Novogene Co., Ltd and sequenced on an Illumina HiSeq1000 system. Differential expression analysis was performed using DESeq2 (version 1.38.3), implemented in R (version 4.2.2) using raw count data as input obtained from Hisat2. Statistically significant genes were identified using threshold of | log2 fold change | >= 1 & adjusted *P* values (multiple testing using the Benjamini–Hochberg method) less than 0.05 (at 95% confidence). All *P* values reported in context of differential expression analysis were generated using the Wald test. Enrichment Analysis was performed with ClusterProfiler (14) software package (version 4.9.0.002) in R to perform functional enrichment for biological pathways using gene sets for pathways from three databases: Hallmark gene sets from The Molecular Signatures Database (MSigDb), Kyoto Encyclopedia of Genes and Genomes and Reactome gene sets downloaded from MSigDB. The enrichment analysis was performed on the ranked list of genes using the DESeq2 test statistic as a ranking metric.

### Antibody generation

Mouse Bsep recombinant protein production (NP_003733.2) with C-terminal His-Strep-Flag tags was carried out by Viva Biotech (Shanghai, China). Antibody production was carried out by Pocono Rabbit Farm (Canadensis, PA). Antibodies were raised in a NZW rabbit, which was bled to measure antibody production by ELISA at 42 and 70 days after first injection. Antisera were prepared and affinity chromatography was carried out using a ligand affinity column prepared according to a standard operating procedure (Pocono Rabbit Farm). The specificity of the polyclonal Bsep antibody was validated by the detection of overexpressed mouse and human BSEP constructs in HEK293T cells, mouse and human primary hepatocytes.

### Western blots ± PNGase

Total protein extracts were prepared from flash-frozen liver tissue by homogenization in RIPA buffer supplemented with a combined protease and phosphatase inhibitor cocktail. Deglycosylation was carried out with Rapid PNGase F (New England BioLabs) for 10 min at 50°C. The resulting product was reduced and denatured in Laemmli sample buffer containing fresh DTT, resolved on 4–12% Bis-Tris gels, transferred to nitrocellulose membranes, immunodetected with antibodies, and imaged using a ChemiDoc system (Bio-Rad).

### Quantitative real-time PCR

Total RNA was extracted from mouse liver homogenates using the QIAzol Lysis Reagent (Qiagen, Catalog No. 79306) followed by purification with the RNeasy Plus Mini Kit (Qiagen, Catalog No. 74136), according to the manufacturer’s instructions. Reverse transcription was performed using the High-Capacity complementary DNA Reverse Transcription Kit (Applied Biosystems, Catalog No. 4368814) with random primers to generate complementary DNA. Each reaction contained TaqMan Gene Expression Master Mix (Applied Biosystems, Catalog No. 4369016) and gene-specific TaqMan primer/probe assays for *Abcb11* (TaqMan Assay # Mm0045168_m1), *Gusb* (TaqMan Assay # Mm01197698_m1), and *Tbp* (TaqMan Assay # Mm01277042_m1). Quantitative real-time PCR reactions were performed in triplicate using a Bio-Rad CFX384 Real-Time PCR Detection System. The expression levels of *Gusb* and *Tbp* were used as internal reference genes for normalization.

### Histological analysis and immunohistochemistry

After euthanasia, all mouse livers underwent transcardial perfusion with ice-cold PBS before removal of the liver. Liver tissues were embedded in optimal cutting temperature compound and frozen. Histological sections of 10 μm thickness were cut and utilized for subsequent staining. For immunohistochemistry, liver section slides underwent deparaffinization and antigen retrieval using Rodent Decloaker (Biocare). To block endogenous peroxidases, sections were incubated in BLOXALL Readymade Solution (Vector Labs) for 15 min. Following a wash with MilliQ water, sections were incubated in Power Block solution (BioGenex) for 15 min, followed by a 1 h incubation with 3% donkey serum diluted in 0.1 M PBS at room temperature. Slides were incubated with primary antibodies overnight at 4°C (1:500 BSEP). Slides were washed three times following overnight incubation in PBS with 0.1% Tween 20 for 5 min each, incubated in Signal Stain Boost immuno histochemistry detection (Cell Signaling) for 45 min, and then washed again. Color development was achieved using Signal Stain DAB substrate (Cell Signaling), followed by counterstaining with hematoxylin. Dehydration was carried out with increasing ethanol concentrations (70–100%), and slides were cleared with xylene. Finally, slides were mounted using Vectamount Permanent Solution (Vector Labs). A separate set of liver tissues were fixed in 10% neutral buffered formalin, processed to paraffin blocks, cut and mounted on slides (4 μm), stained with H&E (StatLab), and evaluated by an American College of Veterinary Pathologists-board certified veterinary pathologist.

### Statistical analysis

GraphPad Prism software was used to analyze data for statistical significance. Outliers were removed by ROUT method and then distribution was determined. One-way ANOVA or Kruskal–Wallis test with Dunn’s or Dunnett’s comparison were performed on studies with 3 or more groups. In comparisons with only two groups, a two-tailed *t* test was used to determine significance. Mixed effects model with Fishers least significant difference multiple comparison was used for longitudinal analysis.

## Results

### Generation of an *ABCB11* variant landscape

**Fig. 1:**
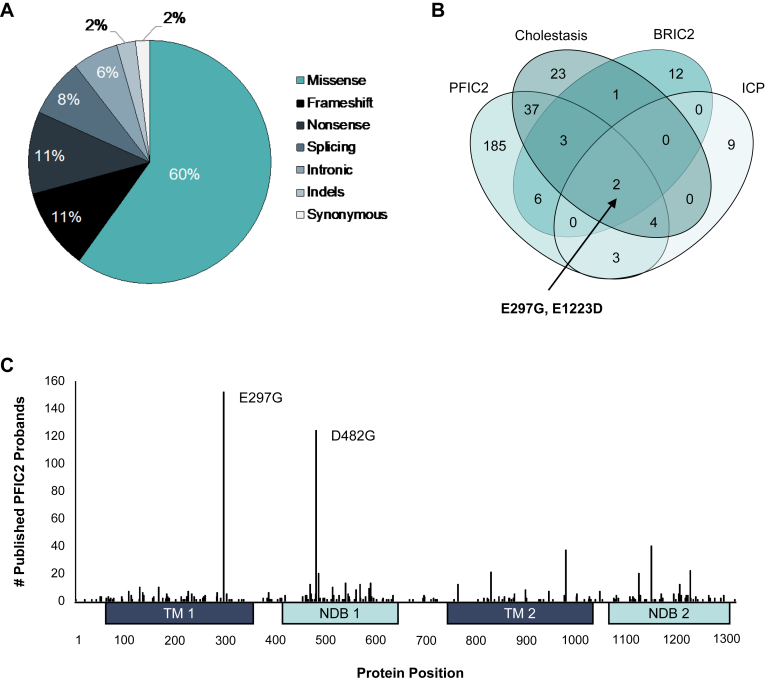
Frequency, position, and associated phenotypes of *ABCB11* missense variants. A: Breakdown by effect type of nonbenign (by ACMG criteria), published *ABCB11* variants that have been observed in a patient with cholestatic disease. B: number of nonbenign, published *ABCB11* missense variants associated with progressive familial intrahepatic cholestasis type 2 (PFIC2), an unspecified/nonsyndromic form of cholestasis, benign recurrent intrahepatic cholestasis type 2 (BRIC2), and/or intrahepatic cholestasis of pregnancy (ICP). C: nonbenign, published *ABCB11* missense variants observed in PFIC2 patients mapped by protein position and number of published, unrelated probands harboring the variant. ACMG, American College of Medical Genetics.

*ABCB11* variants were identified through a comprehensive literature review performed using the Mastermind Genomic Intelligence Platform (10); https://mastermind.genomenon.com/]. A total of 705 published *ABCB11* variants were identified and classified into the 5 tiers of pathogenic (P), likely pathogenic, variant of uncertain significance (VUS), likely benign (LB), and benign (B) according to the ACMG guidelines (12). Out of 705, 476 were nonbenign and observed in patients with PFIC2 or other cholestatic diseases including, benign recurrent intrahepatic cholestasis type 2, ICP, and/or an unspecified/nonsyndromic form of cholestasis (supplemental Table S1). Of these 476 disease-associated variants, 285 (60%) were missense (Fig. 1A), the majority of which were associated with PFIC2 (84%; n = 240) (Fig. 1B). Notably, only two missense variants, E297G and E1223D, were observed in all cholestatic disease states (Fig. 1B).

Of all *ABCB11* variants identified, relatively few have been characterized functionally. Only 61 missense variants had any functional characterization available in the literature (supplemental Table S1). In addition, the methodologies used in these studies vary considerably and has in some cases led to conflicting conclusions regarding the exact impact on BSEP function. This lack of functional characterization contributes significantly to the number of ABCB11 VUS as per ACMG guidelines. Of the 285 *ABCB11* missense variants observed in a patient with cholestatic disease, 219 (75%) have been classified as a VUS based on current evidence available in the literature.

Two pathogenic variants, E297G, located in the transmembrane domain, and D482G, located in the nucleotide binding domain, were significantly more prevalent than other variants, and were observed in 152 and 124 published probands with PFIC2, respectively (Fig. 1C and supplemental Table S1). E297G specifically has also been observed in 7 probands with ICP, 4 probands with an unspecified/nonsyndromic form of cholestasis, and 2 probands with benign recurrent intrahepatic cholestasis type 2 (supplemental Table S1). In vitro analysis of the BSEP E297G protein in cell line systems has consistently demonstrated trafficking deficits from the endoplasmic reticulum, resulting in significantly reduced membrane localization (8, 15, 16, 17, 18). While these biochemical data suggest E297G results in impaired hepatic BA efflux due to decreased canalicular expression, leading to cholestasis, no studies have directly tested this connection. To address this, we sought to generate and validate a knockin mouse model of E297G.

### Generation and genetic validation of Bsep^E297G^ homozygous mice

**Fig. 2:**
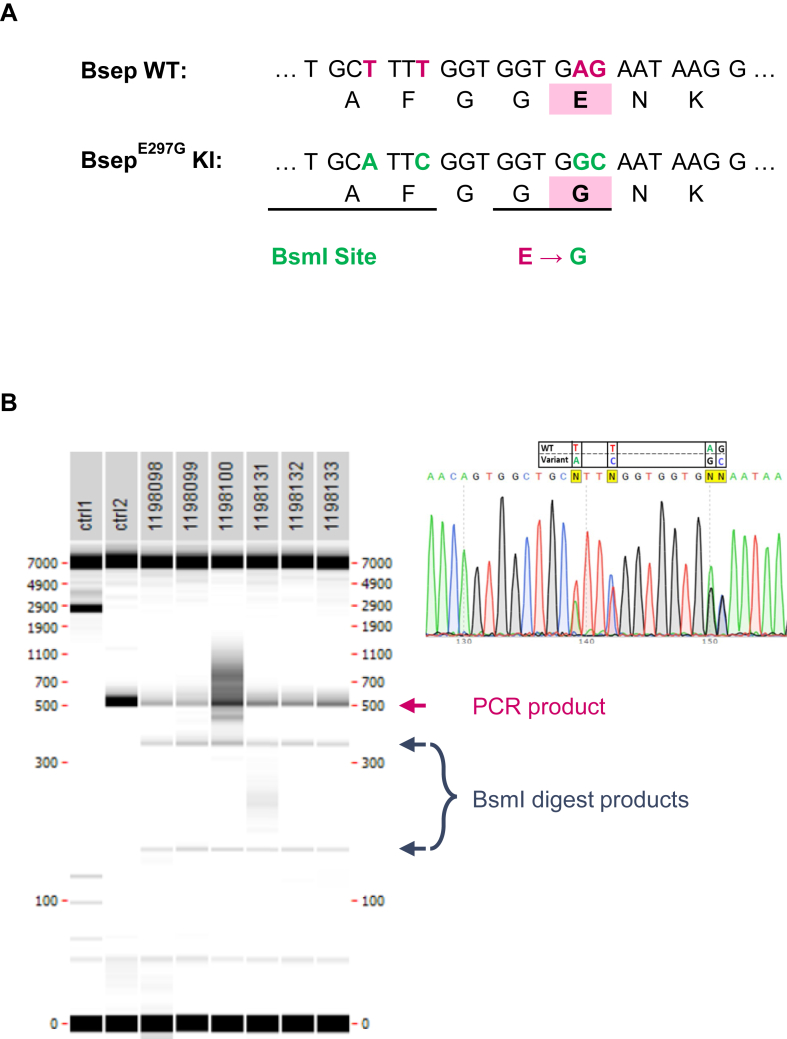
Generation of PFIC2 mouse model by introduction of pathogenic BSEP human missense variant into mouse Bsep. A: Sequence of mouse *Bsep* edited from WT nucleotides (red) to the E297G allele nucleotides containing both the E to G conversion and introduction of BsmI restriction site (green). B: Genotyping gel of F1 HET animals indicating PCR product band and BmsI digest band (top). Sequencing chromatograms demonstrating heterozygosity of the edited nucleotides. BSEP, bile salt export pump; HET, heterozygote; PFIC2, progressive familial intrahepatic cholestasis type 2.

Mouse and human Bsep/BSEP are 82% identical at the amino acid level including the PFIC2-relevant glutamic acid (E) at position 297. The editing strategy utilized pronuclear injection of one-cell stage fertilized C57BL/6NTAc embryos with CRISPR/Cas9, and guide RNA that targeted exon 9 of the endogenous *Abcb11* locus for the introduction of the amino acid glycine (G) at residue 297, and a silent BsmI restriction site (Fig. 2A and supplemental Fig. S1A). Multiple rounds of pronuclear injection yielded 21 F0 founder pups, of which 5 had the appropriate DNA fragments (350 bp and 169 bp) after BsmI digestion of PCR products, indicating these animals had a good chance of harboring the desired sequence (supplemental Fig. S1B). PCR products from genotyping of these animals were subcloned, and 12 colonies were picked from each animal for sequencing. One mouse demonstrated high mosaicism of the edit, indicating a probability of germline transfer. This F0 founder was used in in vitro fertilization, and the resulting progeny demonstrated germline transfer of the BsmI restriction site, as well as of the *Bsep*^*E297G*^ amino acid change (Fig. 2B). Crossing F1 animals to WT C57BL/6NTac generated all genotypes in the expected Mendelian frequency (supplemental Fig. S1C).

### Bsep^E297G^ variant results in impaired protein trafficking

**Fig. 3:**
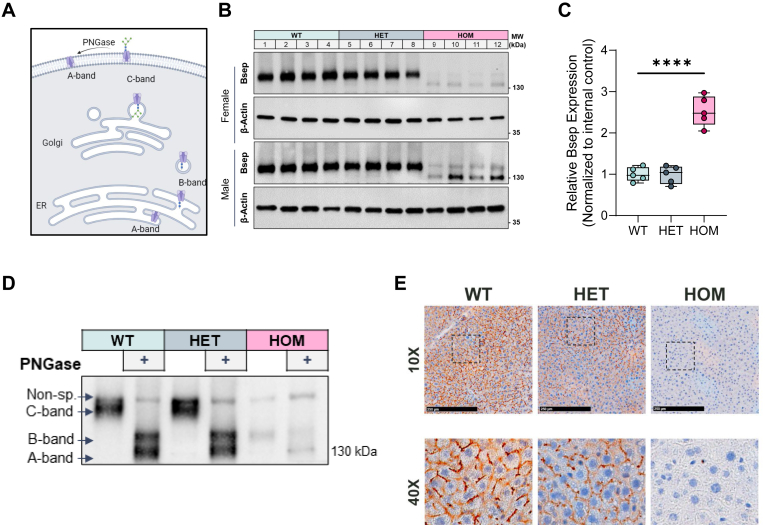
BSEP protein processing is defective in Bsep^E297G^ mice. A: Graphic depiction of BSEP protein maturation and sequential glycosylation in the ER and Golgi apparatus as it traffics to the plasma membrane. B: Western blot of liver lysate from female (top) and male (bottom) WT, HET, and HOM animals indicating the presence of B- and C-band. C: *Bsep* mRNA qPCR analysis from WT, HET, and HOM female mice. D: Western blot of liver lysates from WT, HET, HOM animals subjected to PNGase to generate A- and B-band. E: Immunohistochemistry for Bsep in frozen liver cryo-sections from WT, HET, and HOM mice. BSEP, bile salt export pump; ER, endoplasmic reticulum; HET, heterozygote; HOM, homozygous; qPCR, quantitative real-time PCR.

Posttranslational modification of BSEP with *N*-linked glycosylation is necessary for protein stability, proper surface localization, and function (19). BSEP is first glycosylated in the endoplasmic reticulum and further glycosylated in the Golgi to direct localization to the plasma membrane (Fig. 3A). The glycosylated forms can be distinguished from one another based on their migration in SDS-PAGE. The fully glycosylated mature protein migrates the slowest at 150 kDa (c-band), while the intermediate glycosylated protein migrates farther at 140 kDa (b-band), but not as far as the unglycosylated protein, typically observed at 120 kDa (a-band) (19). Western blot analysis for Bsep in all three mouse genotypes demonstrates the presence of a presumptive lower molecular weight b-band, that is evident in homozygous (HOM) animals, while only the fully glycosylated c-band is observed in WT and heterozygote (HET) liver lysates (Fig. 3B). The reduced levels of Bsep protein in the HOM animals is not due to an effect on transcription, as in fact, the Bsep mRNA levels in HOM animals are increased approximately 2.5-fold relative to mRNA levels in WT and HET animals (Fig. 3C). Treatment with PNGase collapses c-band in WT and HET, as well the lower molecular weight band in HOM to a lower MW, indicating that the lower molecular weight protein identified in HOM is indeed b-band (Fig. 3D). Additionally, the higher molecular weight band observed in HOM mice (Fig. 3B) is confirmed as nonspecific, given its persistence post PNGase treatment. Consistent with the lack of glycosylation and reduction of protein trafficking and stability in HOM mice, canalicular membrane–localized Bsep is not observed in liver sections from adult mice when compared to WT and HET genotypes (Fig. 3E). A similar observation was made in immunocytochemistry studies with primary mouse hepatocytes derived from the three genotypes (supplemental Fig. S2). These data demonstrate the molecular dysfunction associated with the human E297G variant is recapitulated when the same variant is introduced into the mouse gene.

### HOM Bsep^E297G^ mice recapitulate PFIC2 pathophysiology

**Fig. 4:**
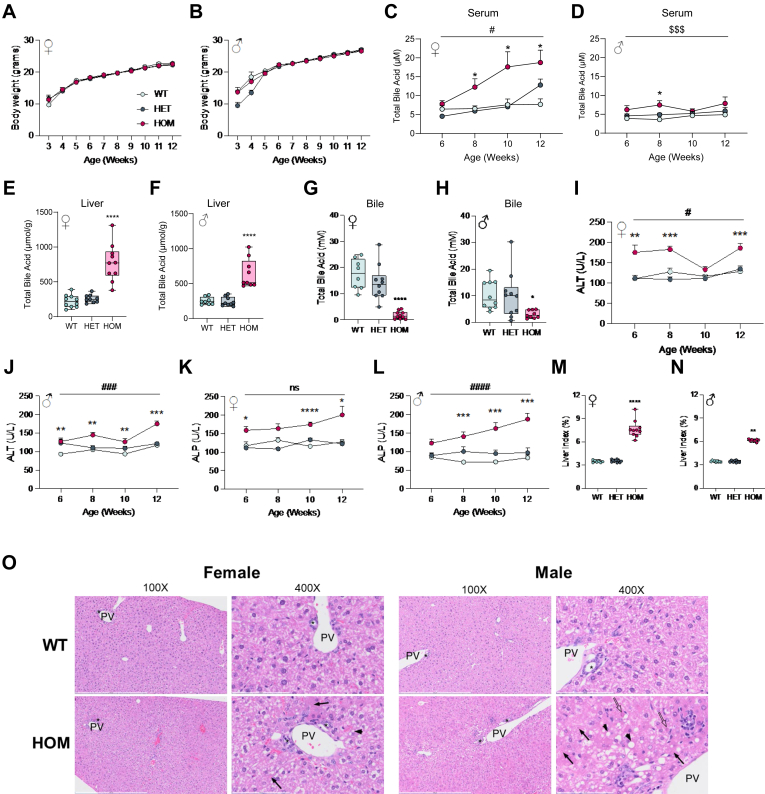
BSEP^E297G^ mice demonstrate evidence of cholestasis and liver injury. A and B: Body weight of female and male WT, HET, and HOM mice from 3 weeks to 12 weeks of age. C and D: Serum BA levels in female and male mice from 6 to 12 weeks of age. E and F: Liver BA levels in female and male mice at 12 weeks of age. G and H: Biliary BA levels in female and male mice at 12 weeks of age. I and J: Serum ALT levels in female and male mice from 6 to 12 weeks of age. K and L: Serum alkaline phosphatase levels in female and male mice from 6 to 12 weeks of age. M and N Liver index in female and male mice at 12 weeks of age. O: H&E staining in male and female WT and HOM at 6 weeks of age (n = 2/group). Black arrows: cytoplasmic inclusions, black arrowheads: lipid-type vacuoles, open arrows: single-cell hepatocyte necrosis/apoptosis, white∗: infiltrating lymphocytes and macrophages, PV: portal vein, ∗: bile duct. ∗∗∗*P* < 0.001, ∗∗∗∗*P* < 0.0001 Fischer’s least significant difference for WT versus HOM, #*P* < 0.01 Mixed effects model for Time x Genotype. $$$ *P* < 0.001 Mixed effects model for Genotype. ALT, alanine aminotransferase; BA, bile salt; BSEP, bile salt export pump; HET, heterozygote; HOM, homozygous.

No difference in body weight between the three genotypes of either sex was observed over 12 weeks (Fig. 4A, B). As compared to WT and HET, HOM mice demonstrate clear evidence of cholestasis from 6 weeks of age. Specifically, female present with an elevation in serum BA content (Fig. 4C), a clinical indicator of PFIC2, and an approximately 4-fold increase in liver BA content at 12 weeks (Fig. 4E) was observed. Moreover, and consistent with Bsep deficiency, biliary BA content was substantially decreased (Fig. 4G). These data indicate that the E297G mutation, and subsequent lack of canalicular localization, leads to reduced BA efflux capacity leading to hepatic accumulation of BA and backflow into the systemic circulation. Male mice presented with a similar pattern (Fig. 4D, F, H); however, the magnitude of change is less, specifically serum BA. This difference, as has been observed of Bsep KO mice (20), indicates sexual dimorphism is also present in the Bsep^E297G^ mouse line.

Hepatic accumulation of BA in HOM mice resulted in the elevation of hepatoxicity biomarkers. Specifically, ALT is significantly elevated in HOM mice compared to WT and HET mice and demonstrates an increase over 12 weeks (Fig. 4I, J). Similarly, ALP is increased as early as 6 weeks in HOM mice compared to WT and HET (Figure 4K, L). Consistent with PFIC2, GGT levels were unchanged in HOM mice (data not shown). Liver index (liver weight as a percentage of body weight), an indicator of hepatomegaly—a cholestatic pathophysiology—doubled in HOM versus WT and HET mice (Fig. 4M, N). Histopathologic analysis of 12-week-old animals demonstrated minimal to mild histopathologic lesions in livers of HOM mice; periportal hepatocytes exhibited single cell necrosis/apoptosis, lipid-type cytoplasmic vacuoles, and cytoplasmic inclusions, with rare infiltration of mononuclear inflammatory cells (lymphocytes, macrophages). Interestingly, findings were slightly more severe in male compared to female HOM mice and were absent in age and sex-matched WT mice (Fig. 4O).

**Fig. 5:**
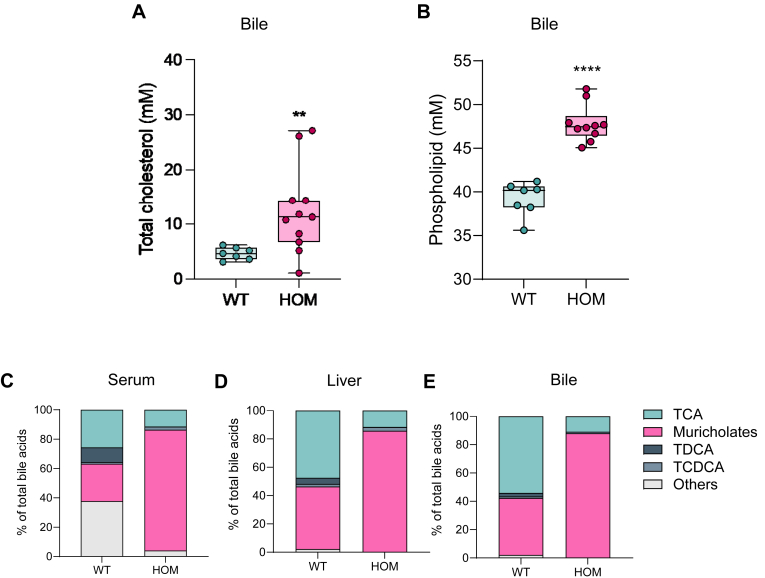
Hepatobiliary bile homeostasis is disrupted in Bsep^E297G^ mice. Composition of bile from the gallbladder of female 12-week-old WT and HOM Bsep^E297G^ mice. A: Bile phospholipids levels and (B) cholesterol levels. BA composition of WT and HOM Bsep^E297G^ mice for (C) serum, (D) liver, and (E) bile. ∗∗*P* < 0.01, ∗∗∗∗*P* < 0.0001 unpaired two-tailed *t* test. BA, bile salt; BSEP, bile salt export pump; HET, heterozygote; HOM, homozygous.

Analysis of bile composition revealed increases in both phospholipid and cholesterol content compared to WT animals (Fig. 5A, B), as is observed in Bsep KO mice (20). Next, we analyzed BA species present in bile using LC-MS/MS. Gallbladder BA composition in HOM animals was altered compared to WT controls. Specifically, muricholates comprised a greater proportion of bile with a corresponding decrease in taurocholic acid and almost complete absence of taurodeoxycholic acid (Fig. 5E), indicating a counter-regulatory shift toward a less toxic BA profile. As expected, both liver and serum BA composition mirrored that observed in the gallbladder (Fig. 5C, D). In sum, these data highlight that the Bsep^E27G^ mouse line recapitulates some of the key core pathophysiology of PFIC2, such as indicators of cholestasis, liver injury, and hepatomegaly, while it also phenocopies many aspects of the Bsep KO line (Table 1).

**Table 1:** Bsep^E297G^ homozygous mice are a translational model of PFIC2

Endpoint	PFIC2	Bsep^E297G^ Homozygote	Bsep^KO^
Cholestasis	✓	✓	✓
Serum BAs	↑	↑	↑
Bile composition	ND	↑ PL; ↑Ch	↑ PL; ↑Ch
Failure to thrive	✓	✗	✗
ALP	↑	↑	↑
ALT	↑	↑	↑
GGT	↔	↔	↔
Hepatomegaly	✓	✓	✓
Liver failure	✓	✗	✗
Pruritus	✓	ND	ND
Sexual dimorphism	✗	✓	✓
Counter-regulatory transcription	ND	✓	✓
Necrosis	✓	✓	✓
Bile duct hyperplasia	✓	✗	✓
IBAT responsive	✓	✓	ND

ALP, alkaline phosphatase; ALT, alanine aminotransferase; BA, bile acid; BSEP, bile salt export pump; Ch, cholesterol; IBAT, ileal bile acid transporter; ND, not determined; PFIC2, progressive familial intrahepatic cholestasis type 2; PL, phospholipid.

### Cholestasis in Bsep^E297G^ mouse model promotes a counter-regulatory transcriptional program

**Fig. 6:**
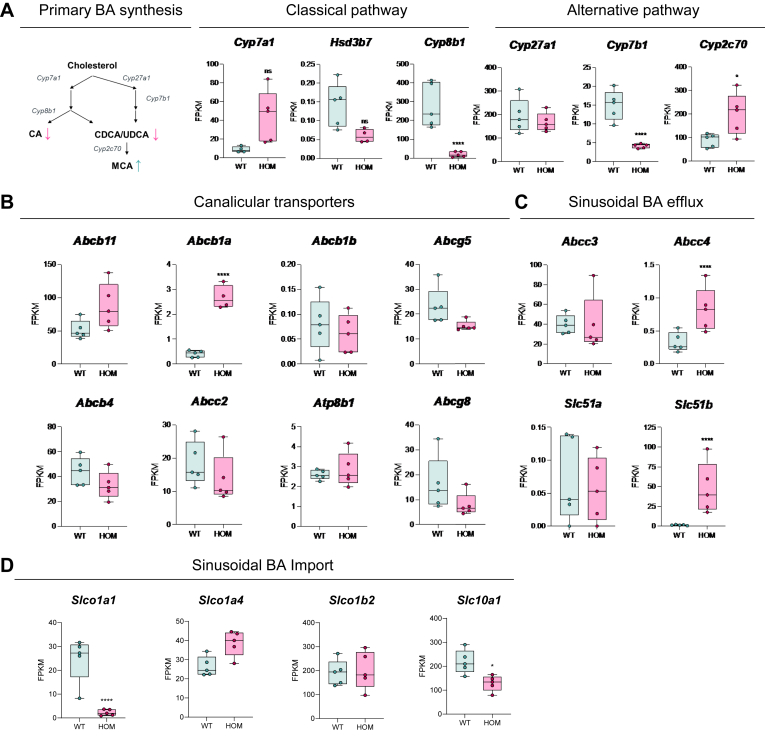
Transcriptomic and pathway analysis of Bsep^E297G^ mice identified altered gene expression profile consistent with a counter-regulatory response to cholestasis. A: Changes in primary BA synthesis pathways in WT and HOM mice. B: Changes in canalicular transporter gene expression in WT and HOM mice. C: Changes in expression of sinusoidal BA efflux genes. D: Changes in expression of sinusoidal BA import genes. ∗*P* < 0.05, ∗∗*P* < 0.001, and ∗∗∗∗*P* < 0.0001 with Benjamini–Hochberg correction. BA, bile salt; BSEP, bile salt export pump; HOM, homozygous.

To understand the molecular consequences of disrupting Bsep protein maturation, we performed RNA-seq on the livers from WT and HOM female mice. Significant changes were observed between genotypes and gene set enrichment analysis with MSigDB Hallmark collection (21) indicated enrichment of pathways consistent with the cholestatic pathophysiology observed in the HOM mice (supplemental Fig. S3A, B). Specifically of note, decreased expression of genes in BA metabolism, peroxisome pathway, and fatty acid metabolism pathways, and increased expression of genes in cholesterol homeostasis pathway were observed. Deeper analysis indicated that while there is no change in Cyp*27a1* and *Hsd3b7* expression, both *Cyp8b1* and *Cyp7b1* were decreased, while *Cyp7a1* and *Cyp2c70* were increased (Fig. 6A middle and right). These gene expression profiles are consistent with, and likely explain the changes in HOM BA composition; decreased *Cyp8b1* and *Cyp7b1* correspond with decreased cholic acid and chenodeoxycholic acid respectively, while increased *Cyp2c70* is consistent with decreased chenodeoxycholic acid and increased muricholic acid (22, 23). This indicates that there is a shift in BA synthesis toward the generation of a less toxic BA pool (Fig. 6A, left panel).

Targeted analysis of the expression levels for genes involved in hepatic BA transport demonstrated significant changes in HOM mice. Expression of *Abcb1a*, a canalicular transporter that can efflux BA (Fig. 6B), and sinusoidal BA efflux transporters *Abcc4* and *Slc51b* are increased in HOM mice (Fig. 6C), while the expression of the sinusoidal BA importers *Slc10a1* (Ntcp) and *Slco1a1* (Oatp1a1) are reduced (Fig. 6D). Combined, these data suggest that the hepatocytes adapted their gene expression pattern to maximize the outward flow of BA.

### Inhibition of BA reuptake treats cholestasis in Bsep^E297G^ mouse model of PFIC2

**Fig. 7:**
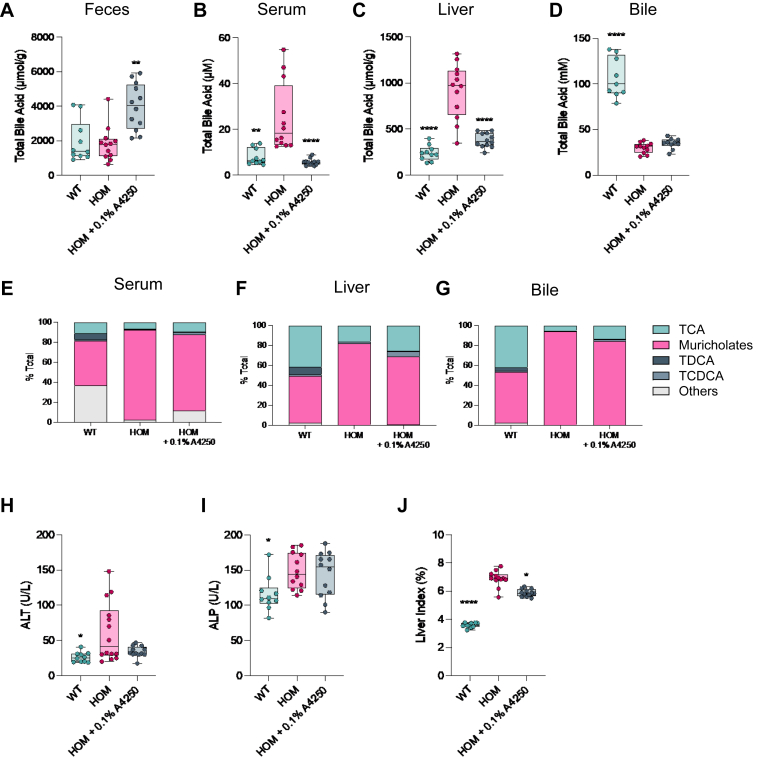
Administration of clinical IBAT inhibitor decreases BA levels in Bsep^E297G^ mice. Fourteen-day treatment of 10- to-14-week-old female HOM Bserp^E297G^ mice with A2450. A: Total BA concentration of feces. B: Total BA concentration of serum. C: Total BA concentration of liver. D: Total BA concentration of bile. BA composition from (E) serum, (F) liver, and (G) bile. H: Serum ALT levels. I: Serum alkaline phosphatase levels. J: Liver index (liver weight to body weight). ∗*P* < 0.05, ∗∗*P* < 0.01, and ∗∗∗∗*P* < 0.0001 one-way ANOVA or Kruskal–Wallis test.ALT, alanine aminotransferase; BA, bile salt; BSEP, bile salt export pump; HOM, homozygous; IBAT, ileal bile acid transporter.

Increasing excretion of BA through IBAT inhibition has demonstrated therapeutic benefit in some PFIC2 patients, including those with a BSEP E297G genotypes. To validate the utility of the Bsep^E297G^ model for translational research, we tested the clinical IBAT inhibitor, A4250 (24) in HOM mice. Consistent with the mechanism of action of IBAT inhibitors, 14 days of A4250 administration resulted in significantly increased fecal excretion of BA (Fig. 7A) and consequential reduction in serum (Fig. 7B) and liver BA (Fig. 7C) concentration, but without impacting biliary BA levels (Fig. 7D). Analysis of BA composition from the same compartments demonstrates a shift toward a WT-like BA composition but not complete normalization (Fig. 7E–G). A4250 treatment reduced ALT (Fig. 7H), but not ALP (Fig. 7I) and improved evidence of hepatomegaly (Fig. 7J). These data demonstrate this model responds faithfully to BA-targeted therapeutics and can serve as a viable translational model for preclinical assessment of novel drug products.

## Discussion

Rare genetic hepatobiliary diseases, such as PFIC2 (*ABCB11*/BSEP deficiency) and progressive familial intrahepatic cholestasis type 3 (PFIC3) (*ABCB4*/MDR3 deficiency) highlight the importance of apposite BA handling in maintaining both liver and biliary health. Thus, the intrinsic toxicity of human BA monomers has necessitated the evolution of protective molecular mechanisms that regulate their transportation and sequestration (25). As the primary BA exporter, BSEP is critical for transporting BA from their site of synthesis in hepatocytes, to their site of biological relevance within the biliary system (26). In humans, genetic BSEP deficiency engenders the rare pediatric cholestatic disease, PFIC2, in which hepatocyte accumulation of BA drives intractable pruritus, failure to thrive and progressive liver injury (3). Treatment of PFIC2-associated pruritus has been revolutionized by the approval of the IBAT inhibitor class of pharmacotherapeutics (27). However, bimodal response rate in clinical trials, to-be-established evidence of disease modification and uncertain long-term efficacy highlight that a significant unmet need remains for PFIC2 patients (28). Recent efforts to understand the genotype-phenotype-IBAT response paradigm in PFIC2 have indicated that disease severity and IBAT efficacy are possibly contingent upon genotype and degree of residual BSEP efflux function (29). Thus, development of novel pharmacotherapeutics requires a comprehensive understanding of *BSEP* genetic landscape and the development of tools to support their preclinical investigation. To this end, here we provide a curated landscape of literature identified, disease relevant *ABCB11* variants with supporting diagnostic and functional data, and a validated novel mouse model of PFIC2 based on one of the most prevalent disease-causing variants.

The *ABCB11* variant landscape represents the most complete dataset of published variants in *ABCB11* to date with a total of 705 variants, 476 of which were nonbenign and observed in patients with cholestatic diseases. This dataset is fully reference-cited and can serve as a complete resource to support evidence-based genetic diagnosis of patients with PFIC2. In addition, it can serve as a foundation for further characterization of the effect of these variants on BSEP function, which has thus far been limited in nature. Further study in this area could significantly reduce the number of VUS and subsequently improve diagnostic rates for this rare disease.

Among all the disease-associated variants identified in *ABCB11*, the most prevalent was E297G, which is consistent with previous studies in European populations (7). Given its high prevalence, the E297G variant has been well described both clinically and functionally but has not previously been translated into a faithful disease model. To support deeper understanding of PFIC2 pathophysiology and drug discovery efforts, we generated a novel disease model based on the E297G variant.

The HOM *Bsep*^*E297G*^ genotype engineered and bred onto a congenic C57BL/6N background faithfully recapitulates the early hallmark molecular and pathophysiological aspects of PFIC2. Molecular characterization of Bsep^E297G^ in exogenous overexpression in vitro systems has indicated that this variant gives rise to a protein trafficking defect that reduces protein stability and membrane localization, while sparing BA transport function (8). Indeed, livers from the Bsep^E297G^ mice demonstrate a loss of mature Bsep protein and therefore absence of canalicular localization. Consistent with a loss of Bsep function, HOM mice display elevated levels of serum and liver BA and the gene expression pattern is changed in a manner that likely attenuates phenotype severity. Expression of hepatic BA importers decreased while sinusoidal BA exporters and Abcb1a increased (Fig. 6). These changes are not sufficient to normalize hepatic BA load; thus, hepatocytes further adapted by shifting expression of primary BA synthesis genes. Cyp8b1 is required for the de novo synthesis of cholic acid (22) and consistent with its decreased expression in HOM animals, taurocholic acid levels are reduced. Muricholates, a less toxic more hydrophilic species of BA, are increased in HOM as are the levels of the mRNA encoding the enzyme responsible for generating muricholates, Cyp2C70 (23). While we did not confirm that the levels of the proteins encoded by the mRNAs assessed in Figure 6 were similarly changed, the matching steady-state changes in BA synthesis mRNAs and their enzymatic products indicate the gene expression changes are likely sufficient to elicit changes in the levels of the enzymes. This is supported by studies that have established a robust correlation between a number of Cyp enzyme transcript levels and their protein and/or enzymatic activity levels (30, 31, 32, 33, 34, 35, 36). Specifically, Cyp7b1 mRNA levels parallel the enzyme’s specific activity in primary rat hepatocytes (31) and changes in Cyp7b1 mRNA correlated with changes in protein levels in mouse liver (32). Similar observations have been reported for Cyp8b1, with correlations shown for its mRNA and enzymatic activity in rat liver and liver microsomes (33, 34), and its mRNA and protein in Cyp2c70^KO^ mice (35). In addition, the study that established Cyp2c70 is the enzyme responsible for the difference between mouse and human BA metabolism exclusively relied on mRNA levels to determine the relative expression levels among the Cyp2c family of enzymes, as well as to assess responsiveness in the levels of Cyp7a1 and Cyp8b1 (36).

Taken together, these data provide validation of both the molecular dysfunction of the E297G variant and a mechanistic explanation for the phenotype of Bsep^E297G^ mice and are the first to do so in a pathophysiologically relevant context. Moreover, as evidenced from another monogenic ABC transporter disease, cystic fibrosis (caused by ABCC7/cystic fibrosis transmembrane conductance regulator) and pharmacological compounds can rescue membrane localization of pathogenic trafficking variants and transform patient care (37). Thus, the Bsep^E297G^ mouse is the first in vivo model that can support the preclinical interrogation of precision therapeutics, similar to the cystic fibrosis transmembrane conductance regulator corrector molecules, for the treatment of PFIC2 through correction of protein trafficking/stability.

PFIC2 patients HOM for E297G or D482G demonstrate a relatively less-severe clinical presentation, with time to liver transplantation significantly longer than in patients with other missense or nonsense variants (4). Moreover, these patients demonstrate a more effective reduction in serum BA following surgical biliary diversion and, concordantly, should have good responses to IBAT therapy (4). The Bsep^E297G^ model presented herein demonstrates a clinically relevant PFIC2 phenotype including cholestasis, as demonstrated by decreased biliary-BA and increased hepatic and serum-BA content, and elevated markers of liver injury, including ALT, ALP, and hepatomegaly. BA composition and gene expression changes likely reflect a counter-regulatory response to cholestasis and are similarly observed in PFIC2 and other cholestatic disease states (20, 38, 39, 40). While we did not assess the Bsep^E297G^ and Bsep^KO^ lines head-to-head, both manifest qualitatively similar early phenotypes including onset at ∼6 weeks of age, changes in BA composition, biochemical markers of cholestasis-induced liver injury, and compensatory gene expression changes including but not limited to some of the BA transporters and synthesis enzymes discussed above (20, 38). Histologically, at the same age both lines present with mild necrosis and inflammation, while bile duct hyperplasia is apparent in just the Bsep^KO^ line. Young PFIC2 patient (<1 year old) biopsies have a similar mild necrotic and bile duct lesions as the mouse, but patients have mild to severe fibrosis, some cirrhosis, and hepatocyte dysplasia (41). These differences in histological presentation and lack of progression toward liver failure, as is observed clinically, is likely a consequence of species differences and a less hepatotoxic BA profile in mice. Humanizing the bile content of this mouse by breeding it onto the background of the Cyp2c70/Cyp12a1 double KO would likely exacerbate the phenotype, similar to what was published with PFIC3; *Abcb4* loss of function on a background of humanized bile more closely resembled the human pathology of PFIC3 (42).

Clinically, reducing serum BA concentration below a threshold of 102 μM post biliary diversion surgery is correlated with native liver survival in PFIC2, and thus serum BA is used as a prognostic biomarker for disease progression (4). Moreover, serum BA level is an endpoint in PFIC2 clinical trials and contributed to the approval of two IBAT inhibitors. These compounds are effective at lowering serum BA levels by inhibiting reuptake of BA from the ileum back into the liver and forcing their excretion in feces (43). As is observed clinically, the treatment of Bsep^E297G^ mice with an IBAT inhibitor resulted in significantly elevated fecal BA concentration leading to a subsequent decrease in serum and liver BA content, with a moderate impact on biochemical markers of liver injury. However, and consistent with the mechanism of IBAT action, biliary BA content was not altered, highlighting a lack of effect on the underlying mechanism of disease. It is possible that 2 weeks duration of treatment may not be sufficient to observe improvements biliary BA content, ALP, or liver index.

Together, these data support the Bsep^E297G^ mouse line as a validated translational model of PFIC2. The phenocopying of key molecular, biochemical, pathophysiological, and pharmacological features of the clinical population in this line supports its use for the preclinical investigation of PFIC2 therapies and predicts good clinical translation. While not investigated here, this model could also support research into the genotype-phenotype correlation in PFIC2 through generation of compound heterozygous genotypes such as Bsep^E297G:KO^ or could be used in a heterozygous state, in combination with exogenous pharmacological or diet stressors, to model other BSEP relevant human pathologies, such as DILI or BRIC.

In sum, we present two translational tools to support further research into BSEP-related primary cholestasis and enable the discovery and development of novel therapeutics to treat high unmet need patient populations, such as PFIC2.

## Data availability

Data not contained here will be shared upon request from corresponding authors.

## Supplemental data

This article contains supplemental data.

## Conflict of interest

The authors declare that they have no conflicts of interest with the contents of this article.
