# Supplementary material for: An ABCB11 variant registry and novel knockin mouse model of PFIC2 based on the clinically relevant ABCB11 E297G variant
Source: J Lipid Res. 2025 Jun 11;66(7):100840. doi: 10.1016/j.jlr.2025.100840 (PMC12273561; doi:10.1016/j.jlr.2025.100840)
Supplement: Supplemental figures [file mmc4.pdf]

Supp. Fig S1

A

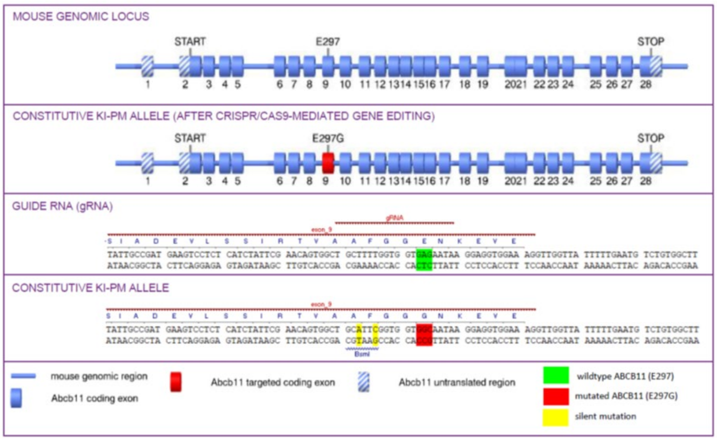

B

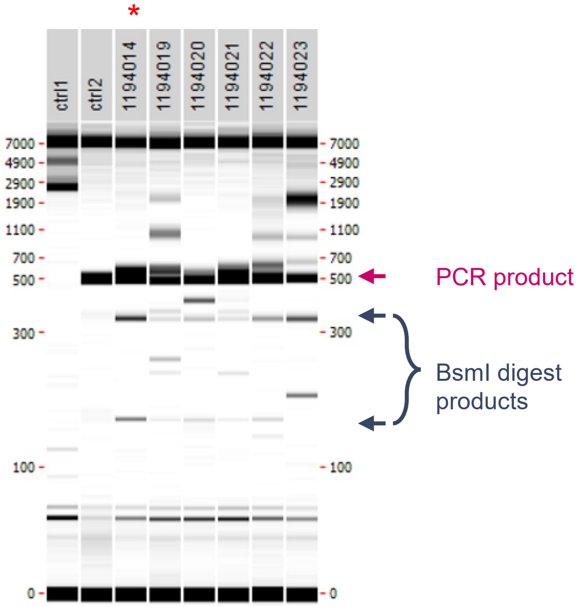

C

HET x HET			
**Genotype**	**Expected**	**Observed**	**% Observed**
**HOM**	40	44	27.5%
**HET**	80	77	48.1%
**WT**	40	39	24.10%
**Total**	160	160	
**χ^2 test**	**0.8779**		

Supp. Fig S2

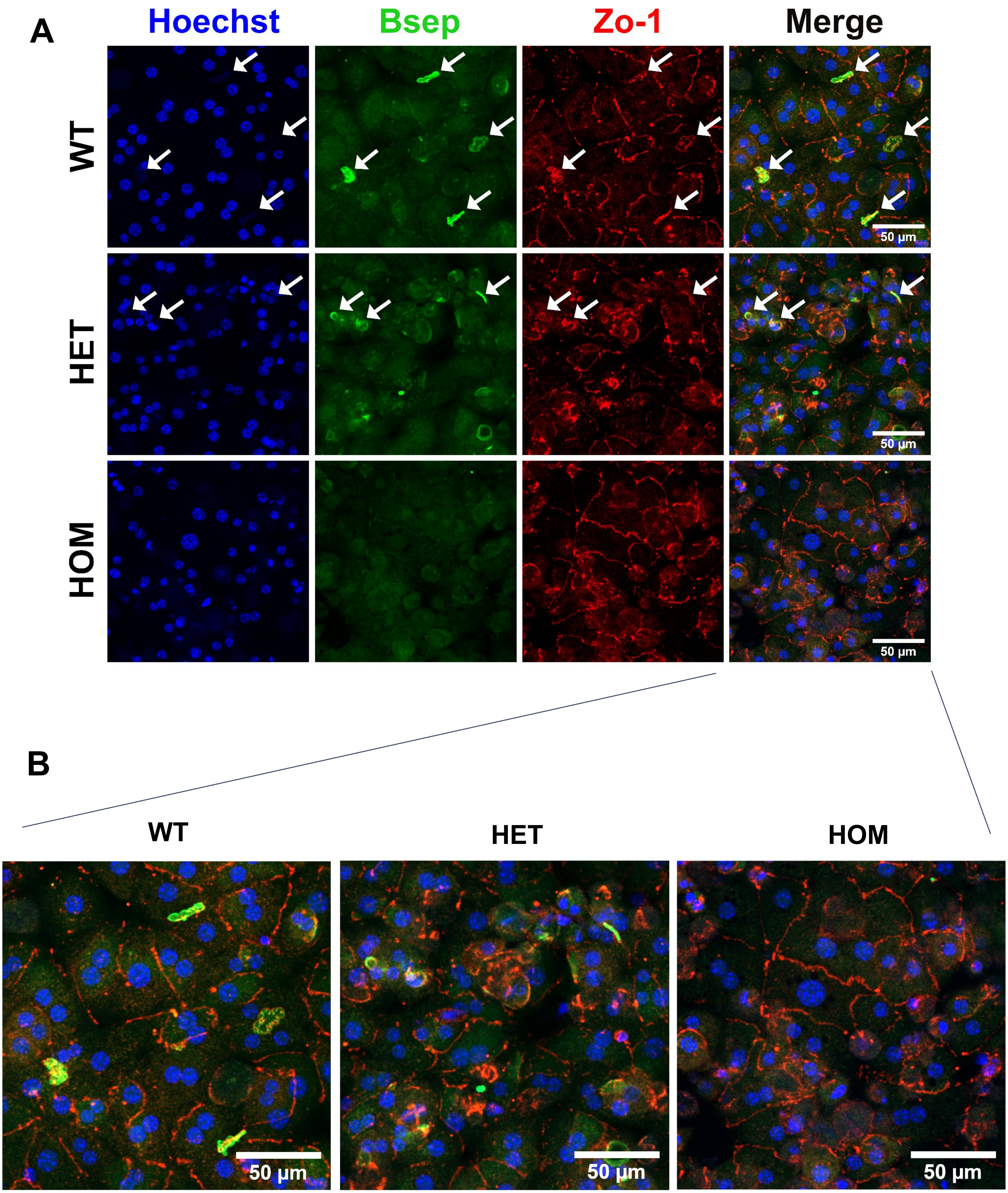

A
Hoechst
Bsep
Zo-1
Merge
WT
HET
HOM
50 µm
50 µm
50 µm
B
WT
HET
HOM
50 µm
50 µm
50 µm

Supp. Fig S3

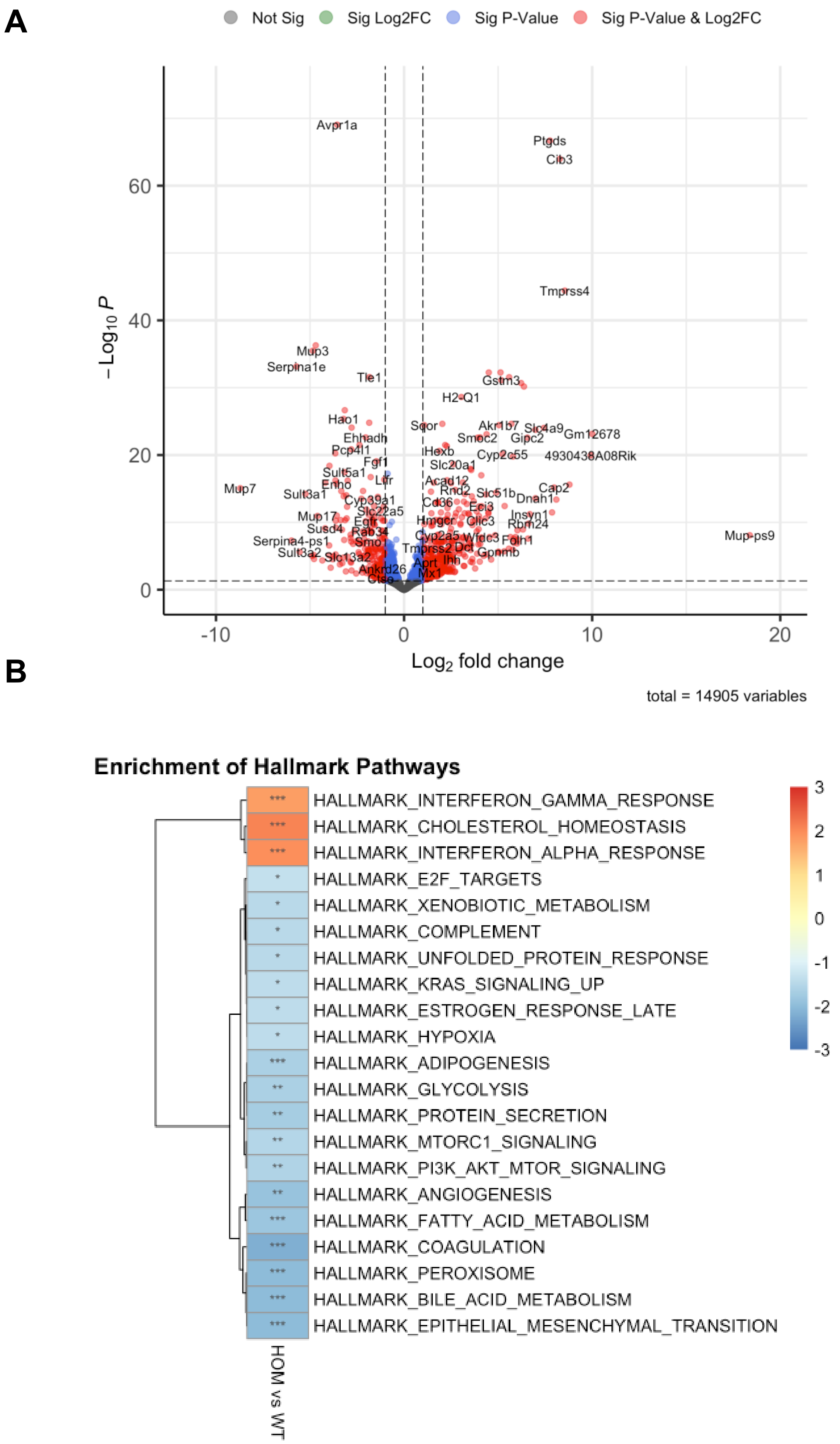

A
Not Sig
Sig Log2FC
Sig P-Value
Sig P-Value & Log2FC
$-Log_{10}$ *P*
Log_2 fold change
total = 14905 variables
Avpr1a
Ptgds
Cib3
Tmprss4
Mup3
Serpina1e
Tle1
Gstm3
H2-Q1
Hao1
Ehhadh
Pcp4l1
Fgf1
Sult5a1
Enho
Ldlr
Mup7
Sult3a1
Cyp39a1
Slc22a5
Mup17
Egfr
Susd4
Rab34
Serpina4-ps1
Smo
Sult3a2
Slc13a2
Ankrd26
Ctse
Sqor
Akr1b7
Slc4a9
Gm12678
Smoc2
Gipc2
Hexb
Cyp2c55
4930438A08Rik
Slc20a1
Acad12
Rnd2
Slc51b
Cap2
Cd36
Eci3
Dnah1
Hmgcr
Clic3
Insyn1
Rbm24
Cyp2a5
Wfdc3
Folh1
Tmprss2
Dct
Gpnmb
Aprt
Ihh
Mx1
Mup-ps9
B
Enrichment of Hallmark Pathways
HALLMARK_INTERFERON_GAMMA_RESPONSE
HALLMARK_CHOLESTEROL_HOMEOSTASIS
HALLMARK_INTERFERON_ALPHA_RESPONSE
HALLMARK_E2F_TARGETS
HALLMARK_XENOBIOTIC_METABOLISM
HALLMARK_COMPLEMENT
HALLMARK_UNFOLDED_PROTEIN_RESPONSE
HALLMARK_KRAS_SIGNALING_UP
HALLMARK_ESTROGEN_RESPONSE_LATE
HALLMARK_HYPOXIA
HALLMARK_ADIPOGENESIS
HALLMARK_GLYCOLYSIS
HALLMARK_PROTEIN_SECRETION
HALLMARK_MTORC1_SIGNALING
HALLMARK_PI3K_AKT_MTOR_SIGNALING
HALLMARK_ANGIOGENESIS
HALLMARK_FATTY_ACID_METABOLISM
HALLMARK_COAGULATION
HALLMARK_PEROXISOME
HALLMARK_BILE_ACID_METABOLISM
HALLMARK_EPITHELIAL_MESENCHYMAL_TRANSITION
HOM vs WT
