# Supplementary material for: An ABCB11 variant registry and novel knockin mouse model of PFIC2 based on the clinically relevant ABCB11 E297G variant
Source: J Lipid Res. 2025 Jun 11;66(7):100840. doi: 10.1016/j.jlr.2025.100840 (PMC12273561; doi:10.1016/j.jlr.2025.100840)
Supplement: Supporting information figure legends [file mmc5.docx]

**Supporting information**

**Materials and Methods**

**Primary mouse hepatocyte isolation and culture**: Primary hepatocytes were harvested from an anesthetized donor mouse by a standard in situ perfusion of the liver using Collagenase IV (Worthington Biochemical) by MB Biosciences. The collected hepatocytes from perfusion were filtered through a 100 µM cell strainer (Corning, USA), centrifuged to collect the cell pellet. The hepatocytes were further purified with Percoll gradients. The purified hepatocytes were suspended in Plating Medium (MB Biosciences) and seeded onto collagen I-coated 24‐well plates at a density of 2-3 × 10^5 /per. The cell plates were cultured in a 37°C incubator with 5% CO2 for 5 hours to allow the cells to completely attach to the plates, then the plating medium was replaced with Hepatocyte Maintenance Medium (MB Biosciences). For a sandwich culture, the seeding medium was replaced with Hepatocyte Maintenance Medium that contains 100 μg/ml collagen I (Sigma). Hepatocytes were allowed to recover overnight, and experiments were started 24 h post isolation.

**Immunofluorescence**: Sandwich-cultured primary mouse hepatocytes were fixed with 4% paraformaldehyde in 0.1 M phosphate-buffered saline (PBS; pH 7.4) for 15 min at room temperature and washed with PBS three times. The fixed cells were blocked in PBS containing 3% bovine serum albumin and 0.2% Triton X-100 (Blocking buffer) for 1 hr at room temperature. Cells were incubated with primary antibodies in the blocking buffer overnight at 4 °C and washed three times in PBS. The primary antibodies used were BSEP (Invitrogen, Cat. PA5-78690, 1:500) and Zo-1 (Invitrogen, Cat. 33-9100, 1:50). This experiment was performed before we generated the custom antibody, therefore that antibody was not used here. Cells were incubated with fluorescence-labeled secondary antibodies, Hoechst33342, and Alexa Fluor 647 Phalloidin (Thermo Fisher Scientific, Cat. A22287, 1:400) in blocking buffer for 1 hr at room temperature and washed three times in PBS. The secondary antibodies used were anti-rabbit Alexa Fluor 488 (Abcam, Cat. ab150077, 1:500) and anti-mouse Alexa Fluor 594 (Abcam, Cat. ab150116, 1:500). After mounting the cells on slide, images were acquired with the Zeiss Cell discoverer 7 with LSM900.

**Figure legends**

**Figure S1. Generation of knock-in mouse model of Bsep^E297G^ mice using pathogenic human variant.**

(A) The strategy to edit exon 9 of mouse Bsep to introduce a missense mutation encoding for a glycine at amino acid 297 denoted by the green (wildtype) and red (mutated) highlighted codon. The guide RNA also contains silent mutations to introduce a BsmI restriction site for genotyping (yellow highlights). (B) Genotyping and BsmI digest from F0 founder mice. Starred lane is the mouse used to generate F1 animals. (C) Outcomes from breeding HET x HET PFIC2 animals.

**Figure S2. BSEP protein localization is defective in HOM mice.**

(A) Immunofluorescence of primary mouse hepatocytes isolated from WT, HET, and HOM mice stained with anti-Bsep (green), anti-ZO-1 (red), and Hoechst (blue). Scale bar, 50 μm white arrows indicate canaliculi formation. (B) Enlarged merged images of WT, HET and HOM mice from (A).

**Figure S3. Analysis of RNA-seq data** (A) Volcano plot showing differential expression of 14905 genes between 12-week-old WT and HOM female animals. Each dot represents one gene. **Blue**: p-value < 0.05 & fold change < 1.5; **Red**: p-value < 0.05 & fold change > 1.5; **Green**: p-value > 0.05 & |fold change| < 1.5. (B) Gene Set Enrichment Analysis (GESA) on RNAseq data from female HOM animals in comparison to female WT Animals. Top up- and down- regulated pathways are shown.

**Table S1. Catalogue of published ABCB11 variants and associated data supporting pathogenicity**

**Table S2. GSEA data for Figure S3**

**Table S3. Differential gene expression data for Figure S3**
